# Quantifying the watercolor effect: from stimulus properties to neural models

**DOI:** 10.3389/fnhum.2014.00805

**Published:** 2014-10-09

**Authors:** Frédéric Devinck, Peggy Gerardin, Michel Dojat, Kenneth Knoblauch

**Affiliations:** ^1^Département de Psychologie, Université Rennes 2, Centre de Recherches en Psychologie, Cognition et Communication, EA 1285Rennes, France; ^2^Institut National de la Santé et de la Recherche Médicale U846, Stem Cell and Brain Research Institute, Department of Integrative NeurosciencesBron, France; ^3^Université Lyon 1Lyon, France; ^4^Institut National de la Santé et de la Recherche Médicale U836Grenoble, France; ^5^Université Grenoble AlpesGrenoble, France

**Keywords:** psychophysics, scaling, signal detection theory, color vision, assimilation, watercolor effect

Visual illusions are perceptions that violate our expectations with respect to what we understand about the physical stimulus, for example, surfaces of identical spectral composition that appear to be of different colors. Such phenomena are thought to reveal mechanisms, biases, priors or strategies that the brain uses to interpret the visual environment. In natural viewing, we perceive all surfaces within a context of surrounding light and nearby objects, and this context affects their appearance. Change in color appearance due to the surrounding light is called color induction and can be of type contrast, when appearance of a test region shifts in chromaticity away from that of the surround and assimilation when the shift is toward that of the surround.

Pinna et al. (Pinna, 1987; Pinna et al., 2001) demonstrated a long-range, color assimilation phenomenon called the Watercolor Effect (WCE) that provides an interesting example for studying such processes. The WCE occurs when a wavy, dark, chromatic contour delineating a figure is flanked on the inside by a lighter chromatic contour on a bright background. The lighter color spreads into the entire enclosed area so that the interior surface is perceived as filled in with a uniform color. Previous studies reported also a weak coloration effect exterior to the contour (Devinck et al., 2005; Cao et al., 2011). The WCE is distinguished from other assimilation illusions due to its spatial extent; the phenomenon has been reported to be observed over distances of up to 45 deg (Pinna et al., 2001). Here, we review and discuss stimulus configurations, based on different procedures, that induce the WCE and their implications for a neural model of this phenomenon.

## Studies of the WCE using hue-cancelation and pair-comparison procedures

In the WCE, the color percept depends on the inner and outer contours. In removing the inner contour, the assimilation effect disappears, and in reversing both contours, the assimilation effect is reversed (some examples are presented in Pinna and Tanca, 2008). Thus, the inducers are considered as a critical feature and previous studies have shown how they modulate the strength of the WCE. Several psychophysical techniques have been used to quantify the specific properties based on different inducer features responsible for the coloration of the WCE. For example, several authors (Devinck et al., 2005; von der Heydt and Pierson, 2006) have used color-matching and hue-cancelation tasks. These methods demonstrate a change in color appearance of the surface enclosed by the double contour, showing a chromaticity shift toward the direction of the inner contour.

Pinna et al. (2001) reported that the coloration effect is stronger and restricted to one side with the use of a dark outer contour. This is crucial to obtain the change in color appearance and it is directly related to the influence of luminance contrast between the two lines enhancing the coloration effect on the inside while containing color spreading to the outside. In using a hue-cancelation task, Devinck et al. (2005) quantified the coloration effect to test the influence of luminance contrast contours. Although the phenomenon still occurs for equiluminant contours, the magnitude of the chromaticity shift increased with increasing luminance contrast between inner and outer contours. Moreover, Pinna et al. (2001) indicated that the WCE persists when dotted lines are used instead of contiguous lines. In using a hue-cancelation procedure, Devinck and Spillmann (2009) reported that the strength of color assimilation decreased when the separation between the inducing contours increased in width and when lateral separation increased between pairs of dots. Thus, both spatial contiguity between and spatial continuity within the inducing contours are important to induce color onto the enclosed central area. Pinna and Reeves (2006) demonstrated that the coloration effect decreases as the number of juxtaposed contours increases. This discoloration effect could be considered as part of a broader class of phenomena related to inference of surface characteristics from contour information.

Another feature related to the inducing contours is the chromatic composition. Some observations of Pinna et al. (2001) indicated that different pairs of colors generated the coloration effect. In using different stimulus chromaticities, Devinck et al. (2005) found a reliable shift in color appearance that closely followed the direction of the inner contour. Specifically, Devinck et al. (2006) demonstrated with the same procedure that the chromatic coordinates of both contours affect the WCE. When the chromaticity coordinates of the inner and outer contours are approximately complementary in color space (the chromatic contrast is relatively large), the coloration effect is stronger in comparison with other color choices. The WCE has mainly been studied with color choices for the contours of “orange” and “purple.” Examining a color diagram, we note that “orange” and “purple” are approximately complementaries, thus, these are reasonable choices to obtain a strong WCE. These studies demonstrated the dependence of the coloration effect on several chromatic and luminance contrasts.

Cao et al. (2011) but also Devinck et al. (2005) noted that the WCE is perceptually salient but has proved difficult to quantify with precision. Thus, previous results showed large variability within and across observers (Devinck et al., 2005; von der Heydt and Pierson, 2006). Cao et al. (2011) claimed that the matching procedure used in a pilot study did not consistently measure the WCE. For this reason, they tried using paired comparisons to study an achromatic version of the WCE. In their experiment, the luminance of the inner contour was fixed while the outer contour was varied between high and low luminance levels. Presented with a pair of stimuli, observers were asked to choose the configuration for which the interior (fill-in) color appeared darker. Results showed that the assimilation effect disappeared when the luminance of the outer contour varied between the luminance of background and the inner contour. Moreover, authors found that the strength of the WCE reach a peak when the outer contour is displayed with a lower luminance level than the inner contour, after which the effect became weaker when the outer contour was too dark. Consequently, the luminance contrast between both contours affects the assimilation effect but not in a linear way. The use of this method allowed an estimation of the WCE strength based on the probability of discriminating the effect.

## Studies of the WCE using scaling methods

Over the last decade, paired-comparison methods have been extended to estimate perceptual scales within a signal detection framework. Two examples are Maximum Likelihood Difference Scaling or MLDS (Maloney and Yang, 2003; Knoblauch and Maloney, 2008, 2012) and Maximum Likelihood Conjoint Measurement or MLCM (Ho et al., 2008; Knoblauch and Maloney, 2012). Both methods use a maximum likelihood criterion to estimate interval scales that best predict observers' choices. These methods appear to be robust indicating, as well, how the sensitivity of the observer varies to each stimulus magnitude. MLDS is useful for scaling a single dimension whereas MLCM may be used to assess the combined effects of several dimensions on appearance.

**Figure 1:**
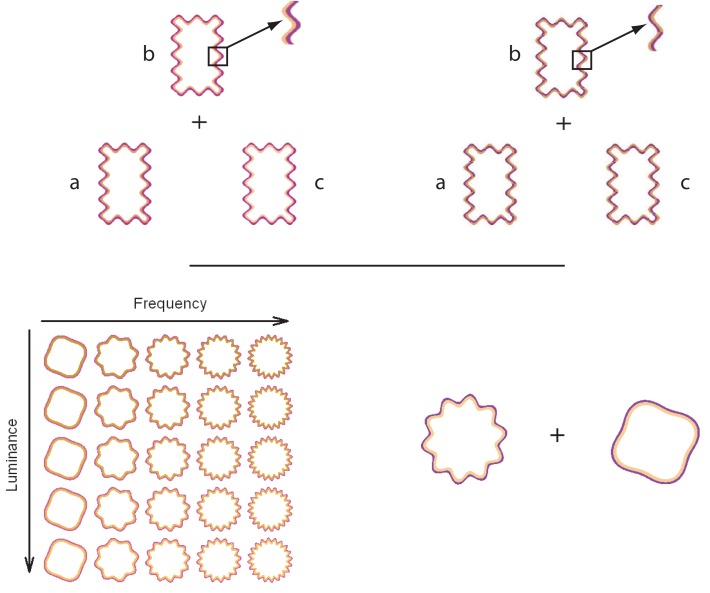
**On the top panels, the triad configuration (a,b,c) for a trial is presented for the WCE (left side) and the braided stimulus (right side)**. This configuration is used during the MLDS experiments. Both panels display a detail of the contour that produced the WCE and the control stimuli in which the two contours are braided. For the control condition, no apparent filling-in occurs. On the bottom panels, an example of stimulus set is displayed on the left side for the MLCM experiment used by Gerardin et al. (2014). The table shows the set of stimuli displayed in the luminance/frequency condition. Each column corresponds to a different luminance elevation and each row to a different frequency. In conjoint measurement experiment, a pair of WCE or braided stimuli was presented from the stimulus sets in each trial. An example of one trial is displayed on the right side and the task was to determine which stimulus of the pair had the most salient color.

Devinck and Knoblauch (2012) used MLDS to measure the strength of the WCE as a function of luminance elevation of the inner contour in DKL color space (Derrington et al., 1984). The WCE was generated with orange interior and purple exterior contours around a rectangular region defined by a wiggly contour. Control stimuli were identical except that the contours were braided and generated little filling-in. An example is displayed in the Figure 1 (top panel). For each trial, an observer was presented with 3 stimuli for which the luminances (a, b, c) of the interior contour differed while the purple contour was fixed at a lower luminance than that of the orange contours as shown in the Figure 1 (top panel). The task was to choose whether the color of the enclosed surface b was more similar to that of a or c. Results supported the hypothesis that the strength of the phenomenon increased with luminance elevation of the inner contour. Then, each observer performed a paired-comparison discrimination task. Results suggested that appearance and discrimination data were mediated by a common signal detection model.

As indicated in the previous section, the coloration effect depends on the characteristics of the contours of the stimulus configuration. Thus, we also applied scaling methods to examine other characteristics of the inducing contours that have not received much attention in previous studies. For example, we used MLDS to examine the influence of contour width on the phenomenon (Devinck et al., 2014). The strength of the WCE was found to be size-tuned with the strongest WCE observed for a contour width of about 15 arcmin. Using the optimal contour width, we also examined the influence of the ratio between the outer and inner contours. The strength of this phenomenon was maximal for equal contour widths, suggesting that balance of widths plays an important role in the WCE.

Gerardin et al. (2014) used MLCM to evaluate the influence of the contour frequency and amplitude, and the luminance of the interior contour on the strength of the effect. In their experiment, circular wiggly contour pairs were presented with two of the dimensions covarying independently (luminance/frequency, luminance/amplitude, and frequency/amplitude). A schematic table of the set of stimuli used in the luminance/frequency condition is presented in the Figure 1 (bottom panel). On each trial, observers judged which member of a pair of stimuli randomly selected from the table evoked the most salient fill-in color, as shown in the Figure 1 (bottom panel). As indicated by the previous studies, the strength increases with luminance of the interior contour. The strength of the phenomenon was nearly independent of the amplitude of the undulation but increased with its frequency up to an asymptotic level. An additive model accounted for the joint contributions of each pair of dimensions. Interestingly, the strength of the luminance effect was comparable to that obtained using MLDS, suggesting that these distinct paradigms measure the same neural process.

In both methods, observer's judgments lead to consistent results within and across observers. The use of a signal detection model leads to a quantification and potentially a direct link with neural responses (Green and Swets, 1966; Tolhurst et al., 1983; Britten et al., 1992). Thus, the last section will discuss constraints imposed on any neural model of the WCE.

## Constraints on neural models based on perceptual scale studies

The WCE presents a significant challenge to any complete model of chromatic assimilation. The coloration effect depends on a conjunction of chromatic and luminance mechanisms of the inner and outer contours. The color of a surface depends on its contrast with nearby surfaces. In the WCE, the assimilation effect is characterized by a spread of color from the inner contour onto the enclosed surface area, suggesting a global effect from local stimulation. One possible explanation is that it involves a filling-in process in which a neuronal mechanism detects the contour and generalizes it beyond the confines of the immediate stimulus. Such a process would likely require multiple levels of processing (Pinna et al., 2001; Pinna and Grossberg, 2005; von der Heydt and Pierson, 2006). Our data based on MLDS and MLCM support such a hierarchical model of the WCE.

Devinck et al. (2014) have shown that the coloration effect depends on a mechanism selective to the spatial frequency and to the ratio of width of the inducing contours. These results indicate that the WCE is selectively tuned and it could be constrained by some properties at an early stage. The size tuning is consistent with color-luminance neurons found in area V1 of the macaque (Shapley and Hawken, 2011). Thus, V1 receptive fields seem to be one of the first step as the substrate for the long-range filling-in phenomenon. Gerardin et al. (2014) also found that the WCE is sensitive to the frequency of the contours. The WCE requires a mechanism that is selective to the curvature of the inducing contours. It should be considered as a second step. Different sites could be candidates as possible neural substrate since selectivity for contour curvature is found in many visual areas. Thus, V3 cells are orientation selective and showed sensitivity to spatial frequency (Gegenfurtner et al., 1997). Moreover, some neurons in the inferior temporal (IT) cortex were tuned to the frequency (Schwartz et al., 1983), as well as most V2 and V4 cells substantially respond to some complex frequency stimuli (Kobatake and Tanaka, 1994; Hegdé and Van Essen, 2000; Roe et al., 2012). These constraints are implemented in the FACADE model based on neurophysiological evidence from neurons in cortical areas V1–V4 (Pinna and Grossberg, 2005). In a hierarchical model, two other steps need to be considered, surface detection then color filling-in.

Studies using perceptual scales have provided an important empirical basis for several types of contour mechanisms generating a long-range filling-in percept. Future investigations should look forward to an advance in our understanding of the filling-in mechanism involved in the WCE, not as separate phenomena in comparison with the inner and outer contours, but a consequence of more global aspects of the WCE processing.

### Conflict of interest statement

The authors declare that the research was conducted in the absence of any commercial or financial relationships that could be construed as a potential conflict of interest.
